# B Cell Tolerance and Targeted Therapies in SLE

**DOI:** 10.3390/jcm12196268

**Published:** 2023-09-28

**Authors:** Ioannis Parodis, Xuan Long, Mikael C. I. Karlsson, Xin Huang

**Affiliations:** 1 Division of Rheumatology, Department of Medicine Solna, Karolinska Institutet, 17177 Stockholm, Sweden; ioannis.parodis@ki.se; 2 Department of Gastroenterology, Dermatology and Rheumatology, Karolinska University Hospital, 17176 Stockholm, Sweden; 3 Department of Rheumatology, Faculty of Medicine and Health, Örebro University, 70281 Örebro, Sweden; 4 Department of Dermatology, Hunan Key Laboratory of Medical Epigenomics, Second Xiangya Hospital, Central South University, Changsha 410011, China; lxxxxcsu@163.com; 5 Department of Microbiology, Tumor and Cell Biology, Karolinska Institutet, 17177 Stockholm, Sweden; mikael.karlsson@ki.se

**Keywords:** SLE, lupus, B cell tolerance, neutrophils, NKT cells

## Abstract

Systemic Lupus Erythematosus (SLE) is a chronic systemic autoimmune disease of high clinical and molecular heterogeneity, and a relapsing-remitting pattern. The disease is currently without cure and more prevalent in women. B cell tolerance and production of autoantibodies are critical mechanisms that drive SLE pathophysiology. However, how the balance of the immune system is broken and how the innate and adaptive immune systems are interacting during lupus-specific autoimmune responses are still largely unknown. Here, we review the latest knowledge on B cell development, maturation, and central versus peripheral tolerance in connection to SLE and treatment options. We also discuss the regulation of B cells by conventional T cells, granulocytes, and unconventional T cells, and how effector B cells exert their functions in SLE. We also discuss mechanisms of action of B cell-targeted therapies, as well as possible future directions based on current knowledge of B cell biology.

## 1. Introduction and Scope

Systemic Lupus Erythematosus (SLE) is a chronic inflammatory autoimmune disease which is more prevalent in females of childbearing age and is characterized by multi-organ involvement [1]. Self-tolerance and autoreactive B cells play important roles in the pathogenesis of SLE. The environmental risk factors connected to apoptosis or clearance of apoptosis debris lead to the breach of self-tolerance, causing pathogenic activation of B cells. As a result of this breach, autoantibodies are produced and accumulate in multiple end-organs, often in immune complex deposits, giving rise to a spectrum of symptoms which include proteinuria, hemocytopenia, joint pain, and swelling, and involvement of the nervous system manifesting as epilepsy or psychosis, for example. This review focuses on the mechanisms of B cell tolerance and current B cell targeted therapies for SLE.

## 2. B Cell Development and Subpopulations

Haematopoiesis is comprised by two major pathways for immune cell development, giving rise to myeloid and lymphoid lineages, respectively. B lymphocytes (B cells) constitute an important component of the lymphoid lineage; they develop in the foetal liver and then the bone marrow, where they assemble their antigen-binding B cell receptor (BCR) and go through negative selection. B cells then migrate to secondary lymphoid organs, where they go through positive selection and acquire affinity maturation of antibody responses. Negative selection depletes self-reactive and polyreactive B cells (whose BCRs bind self-antigens) from the repertoire, whereas positive selection ensures that the BCR is functional. It is, however, important to note that a negative selection of B cells is not always complete, which may result in self-reactive and polyreactive B cells that escape into the periphery, and some of those carry autoreactive properties that later can be found as disease-driving autoantibodies in patients with SLE [2]. Selection continues in secondary lymphoid organs, where B cells arriving from the bone marrow turn from transitional type 1 (T1) to transitional type 2 (T2) B cells. The T2 B cells are activated through BCRs and integrating signals, including those from NF-κB- and B cell-activating factors belonging to the TNF family (BAFF), which are needed for survival and, later, activation and selection. T2 B cells develop into naïve subpopulations that inhabit different microanatomical niches and have varying recirculation patterns [3,4]. The main division is between B1 and B2 B cells. B1 cells derive from foetal liver but can also develop from bone marrow in adults [5]. B1 cells can be further divided into B1a and B1b B cells depending on their phenotype. In mice, there are distinct B1 subsets, but an exact equivalent to these in humans remains to be determined and their existence is still debated [6]. B2 B cells can be divided into follicular B cells (FOBs) and marginal zone B cells (MZBs), and these exist in both mice and humans. Of the B2 B cell subsets, FOBs are the major source for B cells recruited into the germinal centre (GC) reaction during adaptive immune responses. The name of MZBs derives from their location in the marginal zone of the spleen [3]. The marginal zone of the spleen is a crucial region where blood-borne pathogens are sequestered by macro-phages with unique arrays of scavenger molecules. The MZBs reside predominantly in the marginal zone of the spleen and cannot be found as a distinct population in other tissues in mice. In humans, on the other hand, MZBs are more abundant and recirculate through lymphoid organs. The contrast is possibly partly due to the difference in the anatomical composition of the spleen between humans and mice, with the human spleen lacking marginal sinus [7]. Although MZBs belong to the B2 B cell population, they share some features with B1 B cells. The MZBs are larger and more metabolically active, and they can be more easily activated by both antigen specific and non-specific stimuli such as TLR activation. Just like B1 cells, MZBs express more IgM than IgD as their BCR and can produce natural antibodies without going through GC selection and activation [8]. They are also self-renewing and partially generated from naïve B cell precursors and unswitched memory B cells [9,10]. The polyreactive feature of the natural antibody repertoire, which primarily comprises IgM antibodies, is important for immune defence. It is thought to exert effects both in the early immune defence and clearance of apoptotic debris in the circulation and serves therefore as a buffer in the host peripheral immune system. However, as polyreactive antibodies are self-reactive by nature, B cells that produce polyreactive antibodies have been proposed to be a possible source of pathogenic self-reactive antibodies. For instance, the BCRs of MZBs are often more polyreactive or self-reactive in steady states than that of FOBs. However, if simultaneously stimulated with self-antigen and pathogen- and/or damage-associated molecular patterns (PAMPs/DAMPs), they become pathogenic and react to self-tissue or organs. Therefore, they are considered to participate in the initial breach of self-tolerance in autoimmune responses. For example, Mackay et al. found that even though MZBs and B1a B cells induce the production of proinflammatory autoantibodies in BAFF-transgenic (BAFF-Tg) mice that develop an SLE-like phenotype, the depletion of B1a B cells or MZBs does not protect BAFF-Tg mice against disease [11]. This would suggest that FOBs and GCs are required. In addition, the enhanced maturation of FOBs and decreased generation of MZBs by intrinsic factors such as TLR, NF-κB signals, or Y-linked autoimmune accelerator (Yaa) mutation are associated with the production of lupus autoantibodies in murine models [12].

## 3. Central and Peripheral B Cell Tolerance

The vast capacity of the adaptive immune system in recognizing different pathogens comes with a cost of potential autoreactivity. Several mechanisms and multilayers of tolerance have evolved to prevent self-reactivity and the occurrence of autoimmune disease. Central B cell tolerance occurs in the early stages of B cell development in the bone marrow when B cells are not fully matured and only express IgM [13]. B cells develop central tolerance through clonal depletion and receptor revision or receptor editing to avoid self-reactivity [14]. Receptor revision allows immunoglobulin gene recombination and a light chain switch in B cells carrying autoreactive antigen receptors. A large portion of immature B cells go through this process to become functionally unresponsive. At the same time, receptor revision contributes to immune diversity by promoting the use of antibody genes that initially rearrange inefficiently. This is the key mechanism for setting the repertoire of naïve and pre-immune B cells. In patients with primary immunodeficiencies, the mutations affecting central B cell tolerance are mostly coupled with BCR signalling, or with molecules that regulate BCR signalling [15]. Thus, if receptor revision is not regulated properly, more high-affinity autoreactive B cells can escape immune surveillance and be recruited to secondary lymphoid organs to subsequently drive autoimmune disease, including SLE. However, a central tolerance mechanism alone is not sufficient for proper B cell development; later B cells need to be fully activated and regulated in the periphery to acquire the capacity of secreting high-affinity antibodies. This is referred to as peripheral tolerance. The most important mechanism of peripheral tolerance is connected to modification by regulatory T cells. Thus, as is to be expected, primary immunodeficiencies affecting peripheral tolerance are mostly connected to T cell function and the capacity of B cells to interact with T cells. This includes signalling from the T cell receptor (TCR) and co-stimulatory molecules. Co-stimulation is important for B cell activation; in the absence of this, B cells can become anergic, meaning that they are more resistant to activation upon a secondary stimulus. The reason for keeping these potentially pathogenic B cell clones in the immune system remains elusive. One possibility could be that depletion of these B cell clones creates niches in the repertoire, giving rise to failed recognition of viruses and bacteria that may carry similar epitopes. Anergic B cells can indeed be useful; it has been shown that the antibody affinity of anergic B cells matures through somatic hypermutation away from autoreactivity [16]. This was proven in an in vivo model where responses to both self-antigen and related foreign antigen were examined. In this in vivo model, B cells directed towards self-antigens were anergic, but could be activated and mutate away from self-reactivity when exposed to the related foreign antigen. These experiments prove that anergic B cells can be used as a source for specific antibody responses to foreign antigens. They also support the current hypothesis that self-reactivity is allowed in the B cell repertoire because of the potential to fight infections. Still, the reservation of anergic B cells requires precise regulation to avoid the occurrence of human immunodeficiency or autoimmune disease, including SLE.

## 4. Regulation of B Cells by Innate Immune Cells

The different naïve B cell populations including both B1 and B2 populations can all be activated to produce antibodies and/or cytokines as a part of a natural immune response. Even though antigen reactivities are enriched in different naïve B cell populations, it is thought that all naïve B cells can be recruited to a specific response. The activation of B cells can either be T cell dependent or T cell independent. In GCs, follicular dendritic cells (FDCs) could present multimerized repetitive antigens, such as LPS or DNA fragments. B cell responses are induced in the absence of T cells when multimerized FDC antigen and antibody immune complexes (ICs) simultaneously cross-link multiple BCRs. This process also needs a second signal from TLRs to provoke a strong antibody response without producing memory B cells [17].

However, the most potent driving force for B cell activation is T cell dependent. The first step for T cell-dependent immune responses is that naïve B cells present antigenic peptides on MHC that have been taken up via the BCR. This presentation is important for the specificity of the immune response since it ensures that the T cells and B cells are specific for the same antigen, although they bind different epitopes on that antigen. Then, the activated B cells in B cell follicles move to the border of the T cell zone to interact with T cells. The binding of CD40 and CD40L through cell–cell contacts provides a secondary signal for the final activation of B cells. The interaction between T cells and B cells also stimulates the secretion of large amounts of cytokines by T helper (Th) cells, which mediates antibody subclass switching and supports the affinity maturation that occurs in the GC [18]. 

In SLE, autoreactive GCs are central for the autoantibody response and the generation of pathological B cell memory. It has been shown that even a small amount of autoreactive B cells can drive a GC response and work as an engine to support other immune reactivities and promote the epitope spreading that occurs in autoimmune diseases [19]. Several pre-GC checkpoints exist to ensure the correct GC reaction once encountered by antigens. One of these is governed by innate type natural killer T (NKT) cells that allow polyreactive B cells to be activated, while preventing them from entering the GC. NKT cells are T cells selected in the thymus for their recognition of the MHC class I-like molecule CD1d. CD1d is expressed by antigen-presenting cells, including B cells and some activated T cells. Instead of presenting peptides, it presents glycolipids that can be foreign or derived from the self. These self-lipids are produced from cell death and cellular stress responses that could potentially be connected to cell death and autoinflammation [20]. 

BAFF belongs to the tumour necrosis factor (TNF) family and is a survival factor for B cells that sets the threshold for B cell activation. Mice that are transgenic for BAFF have increased numbers of B cells (in particular, T2 and MZBs) and elevated levels of autoantibodies, and they progressively develop nephritis, as well as other autoimmune manifestations [21]. BAFF can be produced by several cell subtypes, including granulocytes such as neutrophils. Neutrophils can boost a natural antibody response through the production of BAFF and in response to inflammation [22]. In SLE patients, BAFF is commonly overexpressed and strongly involved in the pathogenesis of renal involvement [23,24]. Interestingly, BAFF and neutrophil gene signatures are correlated with SLE activity [25]. In addition, neutrophils upregulate CD1d and license NKT cells to regulate B cells and prevent those from entering the GC [26]. This mechanism allows for a boost of beneficial natural antibodies but blocks the entry of potentially dangerous autoreactive B cells to the GC. Thus, there is a newly discovered close connection between inflammation, activation of the innate immune system, and B cell activation [27].

## 5. B Cell Functions in Autoimmunity and SLE

Autoimmune response is initiated when immunological tolerance to self-antigens cannot be maintained. As a result, naïve B cells are activated and differentiate into plasma cells. During this process, B cells switch survival factor dependence from BAFF to its closely related TNF family member, a proliferation-inducing ligand (APRIL) [28]. The activation of B cells leads to a state in which B cells produce specific regulatory cytokines including IL-10, IFNγ, TNF, GM-CSF, IL-6, IL-17, and IL-2 [29]. These cytokines have varying regulatory properties. For example, GM-CSF produced by so-called IRA B cells can promote the production of IL-12 from dendritic cells (DCs) in autoimmune diseases [30]. In SLE, the loss of B cell tolerance is controlled in a cell-intrinsic manner by Toll-like receptors (TLRs) that sense nucleic acids in endosomes. TLR7 drives the extrafollicular B cell response and the GC reaction that is involved in autoantibody production and acceleration of SLE, while TLR9 seems to protect against SLE [31]. SLE is characterized by immature B cells lacking IgD and CD27 (double negative B cells). Sanz et al. found a subset of CXCR5-CD11c+ cells in double-negative B cells representing pre-plasma cells. This group of effector B cells (pre-plasma cells) are predominant in SLE patients with active disease, lupus nephritis, or autoantibodies. The overproduction of pre-plasma cells is induced by unregulated TLR7 [32]. Interestingly, most of the excessively expanded pre-plasma cells also exhibit activation of a T-bet transcriptional network [33]. 

This connects to the newly discovered autoimmunity-associated B cells, also known as age-associated B cells (ABCs), where expression of the transcription factor T-bet has been implicated in autoimmunity and situations of chronic inflammation [34]. ABCs require TLR7 and TLR9 activation as well as Th1-type cytokines for their generation and have been found to produce a repertoire of antibodies that are autoreactive. Also, when depleting ABCs in murine models, the mice were protected from autoimmunity, suggesting a pathogenic role of ABCs in autoimmune diseases [35]. Of the cytokine-producing B cells, the most studied in SLE are the ones producing IL-10, oftentimes called regulatory B cells (Breg) [36]. Breg cells have been shown to be able to regulate autoimmunity and maintain immune homeostasis. Defects in the function or numbers of Bregs have been described in SLE and other autoimmune diseases [37,38,39]. This includes impaired production of IL-10 as well as recruitment of Breg cells in response to TLR9 stimulation of plasmacytoid (p)DCs. However, unlike Treg cells, B cells producing cytokines are thought to differentiate and eventually evolve to plasma cells. It is currently unknown what regulates their cytokine producing phase and how long the regulatory phase can be maintained. There are also no specific transcription factors that have been determined to keep B cells as cytokine producers or Bregs. Some Breg cells also express CD1d and interact with NKT cells. It has been shown that in SLE, NKT cells are dysfunctional and present in low numbers. However, the depletion of B cells with rituximab has been shown to reset the B cell populations to normal levels, including CD1d expression, and this, in turn, normalizes the NKT cell function and restores their numbers [40].

## 6. B Cell Directed Treatment in SLE

In recent years, research on the pathogenesis of SLE has made progress, advancing both diagnostics and investigations using histopathology to unravel cellular and molecular mechanisms underlying the disease. These findings have also resulted in progress regarding SLE drug therapeutics. Earlier SLE diagnosis has not only led to a better disease course in terms of disease activity, but also earlier and more suitable therapeutic interventions that limit organ damage. The systemic treatment for moderate to severe SLE includes steroids, antimalarial drugs, immuno-suppressants, and biological agents. Among new treatments, B cell-directed agents include biologics directly or indirectly targeting B cells, as well as immunomodulators such as synthetic dehydroepiandrosterone and hydroxychloroquine that suppress inflammation in a non-selective fashion.

### 6.1. Anti-Inflammatory and Chemotherapy Treatments and Their Impact on B Cells

Glucocorticoids have potent anti-inflammatory effects that also influence B cell activation. B cells express the glucocorticoid receptor and, thus, these treatments can have a cell intrinsic effect. Targeting this receptor affects several signalling molecules in B cells, including AP 1 and NF κB, which are transcription factors downstream the BCR [41]. Glucocorticoids can also directly mediate apoptosis of B cells. It has been shown that immature B cells are especially sensitive to glucocorticoids [42].

Another immunomodulatory drug that may be considered anti-inflammatory is hydroxychloroquine (HCQ), which inhibits the functions of nucleic acid-sensing TLRs. At the concentrations used in rheumatology, HCQ blocks TLR9 ligation, suppressing B cell differentiation into plasmablasts and, hence, IgG secretion [43]. Importantly, antimalarial agents are recommended for all patients with SLE, unless contraindicated, with HCQ being the drug of choice within this drug class, owing to, in relative terms, a more favourable safety profile [44]. Mycophenolate mofetil (MMF) is a derivative of mycophenolic acid, which is an inhibitor of inosine-5′-monophosphate dehydrogenase. Through depleting guanosine and deoxyguanosine nucleotides in T and B cells, MMF blocks lymphocyte proliferation/differentiation and the production of immunoglobins [45]. Cyclophosphamide (CYC) is an alkylating agent; its active metabolites are acrolein and phosphoramide mustard. The cytotoxicity of CYC is caused by DNA cross linking, ultimately resulting in apoptosis of cells undergoing division. When comparing the effects of CYC and MMF on treating SLE, it was found that both drugs influenced disease activity and B cell numbers, with CYC being more efficient in depleting naïve B cells and pre-switched memory B cells [46].

Moreover, drugs that primarily target T cells also exert indirect effects on B cell activation. One such drug is cyclosporine A (CsA), which is a calcineurin inhibitor and cytochrome P450 inhibitor. While the mechanism of action of CsA is mostly related to T cell suppression, e.g., by inhibiting IL-2 production by DCs, impeding T cell proliferation [47], CsA has also been shown to have effects on B cells and B cell migration [48,49]. CsA inhibits IL-2, IL-6, and IFNγ production by CD4+ T cells; especially through the inhibitory effects of IL-4 and IL-6, CsA indirectly inhibits the growth and differentiation of B cells, and the production of immunoglobulins: Refs. [50,51]. Another calcineurin inhibitor that is used in SLE, mainly in the context of lupus nephritis, is tacrolimus, which has tested in multiple trials with encouraging results, especially in Asian LN populations [52]. A reduction in the percentage of immature B cells was observed when PBMCs were incubated with tacrolimus in vitro [53]. The influence of tacrolimus to B cell maturation and antibody response was indirectly assisted by T cells [54,55]. While CsA and tacrolimus may be considered established drugs for the treatment of SLE and LN, voclosporin was recently approved for the treatment of active lupus nephritis, in combination with MMF, after a successful phase III randomized clinical trial. Voclosporin is a new generation, more potent, and more stable calcineurin inhibitor, whose metabolites are more quickly eliminated, adding ease of surveillance to the advantages of voclosporin over CsA and tacrolimus.

Another drug with immunosuppressive effects is methotrexate (MTX), an antifolate antimetabolite that inhibits DNA synthesis. The resulting anti-inflammatory effect is mediated by an accumulation of adenosine that inhibits T cell activation and, in turn, B cells. In connection to autoimmunity, MTX and CYC combined as a treatment of arthritis in mice lead to changes in cellular numbers in lymph nodes and the spleen, including a decrease in Breg and DC numbers [56]. Lastly, azathioprine also constitutes a traditional non-targeted immunosuppressant that is widely used in SLE [44], acting after conversion to 6-mercaptopurine (6-MP), an immunosuppressant prodrug. Among multiple uses in SLE, azathioprine is used as a remission maintenance treatment for LN and is considered a safe drug during pregnancy.

### 6.2. B Cell Suppressing/Depleting Therapies

#### 6.2.1. Anti-BAFF/APRIL

B lymphocyte stimulator (BLyS, also known as BAFF) is produced by antigen-presenting cells, neutrophils, activated T cells, and endothelial cells. BAFF has important properties for normal B cell differentiation, maturation, and antibody production [57,58]. BAFF provides essential signals for B cell activation and survival via the NF-κB and MAPK pathways, mediated by three receptors: the BAFF receptor (BAFF-R), transmembrane activator and calcium modulator cyclophilin ligand interactor (TACI), and B cell maturation antigen (BCMA). BAFF-R ligation provides key signals, while TACI and BCMA also bind APRIL [28]. In 2000, it was found that overactivation of BAFF may bypass T cell surveillance in negative selection. BAFF transgenic mice exerted lupus-like features, including proteinuria, nephritis, and high levels of anti-dsDNA autoantibodies, making this cytokine an attractive target for the treatment of SLE [59,60]. Blocking the BAFF/APRIL signal with a recombinant fusion protein harbouring TACI in SLE mouse models (MRL/lpr and NZB×NZW) ameliorated lupus-like phenotypes [61]. Also, in patients with SLE, there are higher BAFF and APRIL levels and the BAFF/BAFFR is correlated with disease activity [62]. All these findings have encouraged further investigations into the BAFF and APRIL antagonists seeking to treat SLE. In early phase I and II clinical trials, belimumab, a fully human monoclonal antibody targeting BAFF, was proven to be effective and displayed a favourable safety profile [63]. In two subsequent phase III, multicentre, randomized, placebo-controlled clinical trials (RCTs) of active SLE, a dosage of 10 mg/kg belimumab administered intravenously every fourth week plus standard therapy was superior to standard therapy in inducing responses, according to the SLE Responder Index 4 (SRI-4) criteria at week 52 in both trials (Table 1) [64,65]. A decrease of more than 50% and 43% circulating CD20+ B cells and plasma cells was observed in the belimumab-treated groups in these studies [66]. Upon proven efficacy in these two pivotal phase III RCTs, belimumab became the first biological drug to be approved by the EMA and FDA for SLE, after more than 50 years with no trial successes before. Pooled data analysis from the BLISS trials showed that hypocomplementemia and anti-dsDNA positivity, as well as high disease activity, were baseline predictors of response to belimumab [67]. Lupus nephritis (LN) affects up to 60% of SLE patients and is, along with cardiovascular disease, the most common reason for mortality in SLE. LN most commonly occurs early during the course of SLE and is often the manifestation that results in diagnosis [68]. The combination of belimumab and other immunosuppressants has shown promising results in treating some severe subtypes of LN [69]. In addition, the sub-group analysis of the BLISS trial showed improvements of LN, leading to BLISS-LN, a phase III multinational RCT that enrolled 448 patients with active LN (Table 1) [70]. At week 104, 43% of the patients who received belimumab (10 mg/kg) on top of standard induction therapy with glucocorticoids and MMF or CYC met the trial primary endpoint (primary efficacy renal response), compared with 32% in the group of patients who received standard induction therapy along with placebo, thus indicating the efficacy of belimumab in treating patients with active LN and resulting in its official approval by regulatory agencies also for this indication.

In parallel with the development of the BAFF antagonist, a soluble BAFF receptor fusion protein, TACI-Ig, was also developed which neutralizes both BAFF and APRIL, known as atacicept. As BAFF and APRIL share two receptors, TACI and BCMA, and tend to form heterotrimers in circulation, both cytokines are targeted with this approach. As APRIL provides the signal for the survival of plasmablasts and plasma cells, atacicept exerts substantial neutralizing effects. In the 24-week multicentre, randomized, double-blinded, placebo-controlled phase IIb study of atacicept (ADDRESS II trial) with a total of 306 patients included, significantly more patients receiving atacicept 75 mg or 150 mg subcutaneously achieved an SRI-4 response at the endpoint compared with the placebo group (Table 1) [71]. The trial, however, did not meet the primary outcome. Recently, telitacicept, or RC18, which is another fusion protein comprising a recombinant TACI receptor, fused to the Fc domain of human IgG [72]. Patients in the telitacicept group exhibited a greater SRI-4 frequency at week 48 as a measure of improved disease. Telitacicept has now been approved by the National Medical Products Administration (MNPA) for the treatment of patients with SLE in China (Table 1) [73]. The Phase II clinical trial of telitacicept in the United States is ongoing.

**Table 1:** A selection of clinical trials and their endpoints of B cell suppressing/depleting therapies for SLE.

Targets	Biologicals	Trial/Phase	Country	Number Enrolled	Primary Outcome	Result
BAFF	Belimumab	BLISS-76, Phase III (NCT00410384)	136 centres primarily in 19 countries in North America and Europe.	819	SRI-4 at week 52.	Met the primary outcome [64].
BAFF	Belimumab	BLISS-52, Phase III (NCT00424476)	Multicentres in Latin America, Asia-Pacific, and Eastern Europe.	867	SRI-4 at week 52.	Met the primary outcome [65].
BAFF	Belimumab	BLISS-LN, Phase III (NCT01639339)	107 centres in 21 countries.	448	Renal response at week 104.	Met the primary outcome [70].
BAFF/APRIL	Atacicept	ADDRESS II, Phase IIb (NCT01972568)	Multicentres in Europe, Asia, North America, Central and South America.	306	SRI-4 at week 24.	Did not meet primary outcome but reduction in disease activity and severe flares was observed [71].
BAFF/APRIL	Telitacicept	Phase III	Multicentres in China.	249	SRI-4 at week 48.	Met the primary outcome [72].
CD20	Rituximab	EXPLORER nonrenal trial, Phase II/III (NCT00137969)	55 centres in North America.	257	Major/partial BILAG response at week 52.	Did not meet the primary outcome. Reduced risk and frequency of SLE flares was observed [74].
CD20	Rituximab	LUNAR renal trial, Phase III (NCT00282347)	Multicentres in US and Latin America.	144	Renal response using predefined parameters at week 52.	Did not meet the primary outcome. Responders had reduction in dsDNA and C3/C4 levels [75].
CD20	Ocrelizumab	Phase III (NCT00626197)	123 centres in 23 countries in Latin America, Asia, Western Europe, Eastern Europe, US, Canada, and Africa.	381	Renal response rates at week 48.	Initial results suggested some efficacy in treating LN in seropositive patients. Phase III trial was terminated prematurely due to inefficacy [76].
CD20	Obinutuzumab	Phase II (NCT02550652)	46 sites in 12 countries in USA, South America and Europe.	125	Complete Renal Response at Week 52.	Met the primary outcome [77].
CD22	Epratuzumab	ALLEVIATE-1 (SL0003; NCT00111306) and ALLEVIATE-2 (SL0004; NCT00383214), Phase III	ALLEVIATE-1: 16 centres in Europe, UK and USA. ALLEVIATE-2: 28 centres in Europe, UK and USA.	36/54	The revised primary endpoint was BILAG response with no treatment failure at week 12.	Discontinued prematurely due to interruption in supply of medication [78].
CD22	Epratuzumab	EMBODY 1 (NCT0 1262365) and EMBODY -2 (NCT01261793), Phase III	USA, Brazil, and Europe.	793/791	BICLA response rates at week 48.	Did not meet the primary outcome [79].
CD38	Daratumumab	Case serious	USA.	2	N/A	Induced substantial clinical responses in two active SLE patients [80].

BAFF: B-cell activating factor; BICLA: BILAG-based Composite Lupus Assessment; BILAG: British Isles Lupus Assessment Group; dsDNA: double stranded DNA; LN: lupus nephritis; SLE: systemic lupus erythematosus; SRI-4: SLE Responder Index 4.

#### 6.2.2. Anti-CD20

Even though B cells are the source of autoantibodies in SLE, the investigation of B-cell depletion has not been as successful as the anti BAFF/APRIL targeted therapy. Rituximab, a mouse-human chimeric monoclonal antibody that targets CD20, depletes circulating mature B cells and B cell precursors while preserving plasma cells. In two large RCTs, i.e., the extra-renal EXPLORER trial [74] and the lupus nephritis LUNAR trial [75], rituximab added to standard therapy failed to show efficacy at week 52 as per the primary trial endpoints (Table 1). However, add-on rituximab induced greater reductions in proteinuria than MMF alone and greater improvements in anti-dsDNA autoantibody titers, as well as C3 and C4 levels. Consistent with the EXPLORER and LUNAR trials, prospective cohorts also indicated that B-cell depletion may be effective in serologically active SLE patients or patients with refractory LN [81]. In a prospective cohort study, a steroid-free treatment regimen which incorporated rituximab and MMF, showed promising results for patients with lupus nephritis. While the beneficial effects of rituximab have not been apparent for the treatment of lupus, the drug was approved by the FDA for treating non-Hodgkin B cell lymphomas and has been used off-label to treat pemphigus, which is another autoimmune disease characterized by the production of Dsg1 and Dsg3 autoantibodies. Ocrelizumab, another humanized monoclonal antibody against CD20, showed promise regarding efficacy in treating LN in a phase II clinical trial, but the phase III clinical trials were terminated prematurely because of serious infections or inefficacy (Table 1) [76]. Two other human anti-CD20 monoclonal antibodies, obinutuzumab [82] and ofatumumab [83], were investigated further for the treatment of SLE (Table 1). Obinutuzumab showed promising results in patients with proliferative lupus nephritis in a recently published phase II randomized, double-blind, placebo-controlled trial [77]. A possible explanation for why some patients failed in B cell depletion therapy by anti-CD20 antibodies is that the surge of BAFF upon the depletion of B cells with rituximab may contribute to the reconstitution of B cells and, therefore, result in the inability to have a sustained response to the drug [84,85,86]. An excessive formation and impaired degradation of neutrophil extracellular traps (NETs) was also observed in SLE, which could further drive disease. In the Synbiose study, which was a phase 2 open-label single-arm proof of concept study, treatment with rituximab followed by belimumab resulted in a reduction in serological abnormalities and NET formation, as well as significant clinical remissions in patients with severe refractory SLE [87]. However, in the CALIBRATE trial, patients with refractory LN did not benefit from the combination of rituximab, cyclophosphamide, and glucocorticoids followed by belimumab, compared with placebo at 24 weeks [88]. Whether a combination of biologics would benefit patients with SLE or LN still needs to be determined. Also, the connection between neutrophil activation and their production of BAFF and regulation of B cells needs to be explored further.

#### 6.2.3. Anti-CD22

The CD22 receptor is a negative regulator of B cell activation and binds sialic acid glycosylation moieties on the BCR to block activation and migration. Epratuzumab is a humanized monoclonal antibody that targets CD22. It could rapidly decrease CD22 and slightly inhibit other B cell surface markers such as CD19, CD21, and CD79b, and partially deplete B cells. In a phase I, open-label clinical trial with 14 patients with SLE, all patients who received epratuzumab had an improvement in British Isles Lupus Activity Group (BILAG) scores at some point during the study [89]. Even though epratuzumab showed an adequate safety profile and was well tolerated in the subsequent multicentre phase II/III studies, this did not meet the primary efficacy endpoints (phase II: ALLEVIATE-1 and ALLEVIATE -2, including an open label extension study SL0006; phase III: EMBODY 1 and EMBODY 2). Two randomized, double blind, placebo-controlled, multicentre studies (ALLEVIATE-1 and ALLEVIATE-2) were stopped prematurely due to the interruption in availability of the drug (Table 1). In addition, two other phase III multicentre studies, the EMBODY 1 and EMBODY 2 trials, did not meet their primary efficacy endpoint, which was defined as improvement at week 48 based on the BILAG-based Combined Lupus Assessment (BICLA) (Table 1) [78,79].

#### 6.2.4. Anti-CD38

CD38 is widely expressed on T cells, plasma cells, and plasmablasts. It is also expressed by several non-immune cells in the central nervous system, musculoskeletal system, respiratory mucosa, and pancreas. Sancho and colleagues found that the expression of CD38 in T cells is significantly higher in SLE patients compared with controls, and positively correlated with circulating inflammatory cytokines. In addition, the levels of CD38 autoantibodies have been found to be increased in SLE patients with inactive disease. The elevated level of CD38 autoantibodies is correlated with a decreased expression of CD38 and increased plasma level of IL-10, a cytokine that is known to be immunosuppressive [90]. Since autoantibody-producing plasma cells play pivotal roles in the pathogenesis of SLE, targeting CD38 to deplete antibody-producing plasma cells and autoreactive T cells is theoretically viable for treating patients with SLE. Further, daratumumab is a human monoclonal antibody that effectively depletes CD38-positive plasma cells and plasmablasts. It displayed significant efficiency in treating patients with multiple myeloma [91]. Ostendorf and colleagues reported significant clinical improvements in two patients with severe SLE after combined treatment with daratumumab and, subsequently, belimumab (Table 1). A significant decrease in long-lived plasma cells, as well as type I interferon activity and T-cell mediated chronic inflammation, was observed in those patients [80]. At the same time, anti-CD38 antibody therapy has been explored as an alternative treatment option for other systemic autoimmune diseases, including systemic sclerosis, Sjögren’s syndrome, and anti-neutrophil cytoplasmic antibody-associated vasculitis [92]. However, the wide expression profile of CD38 remains a concern for the further application of anti-CD38 monoclonal antibody treatment due to lack of specificity for B cells. Depleting plasma cells with the proteasome inhibitor bortezomib has shown promise in treating serious cases of refractory SLE [93,94].

### 6.3. B Cell Signalling Targeted Therapies

**Figure 1:**
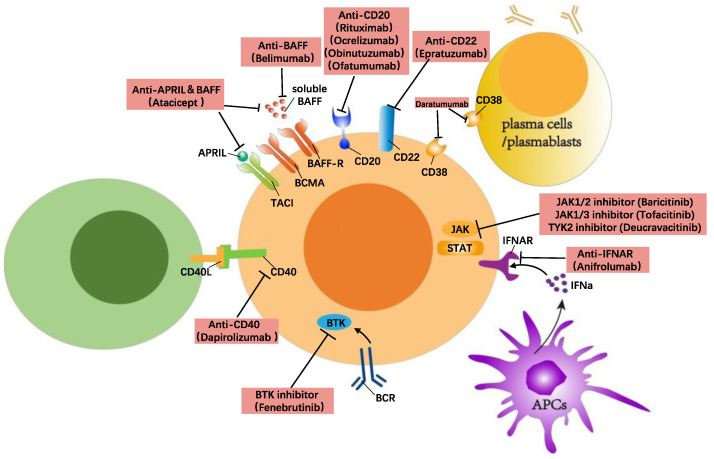
B cell-directed treatment in SLE.

Another method of targeting B cells to treat SLE is blocking the signalling transduction needed for B cell activation. This can be achieved through inhibiting the signalling from either the BCR, co-stimulatory molecules, or survival factors such as BAFF, CD40, and TLRs. Currently, these targets include CD40/CD40L, the Bruton’s tyrosine kinase (BTK), Janus kinase (JAK), Interferon-α (IFNα), NF-κB-inducing kinase (NIK), and Pin-1 (Figure 1).

#### 6.3.1. Anti-CD40/CD40L

The co-stimulatory receptor CD40 belongs to the TNF family and is expressed on various immune and non-immune cells, including B cells, plasma cells, macrophages, DCs, and endothelial cells [95]. CD40 binds to CD40L which is expressed on CD4+/CD8+ T cells and is important for T cell-induced activation during antigen presentation. The antigen presentation by B cells to T cells is a key event and activation via CD40 stimulation leads to proliferation, differentiation, and immunoglobulin production [96]. In mice using the NZB/NZW and MRL/lpr models of lupus, treatment with a CD40 antagonist reversed proteinuria after disease onset and normalized kidney pathology while blocking autoreactive B cell activation [97]. In humans, the antibody dapirolizumab was developed to have high and specific affinity for CD40L. This antibody was used in a phase II, randomized, placebo-controlled trial for SLE (Table 2). Although the trial did not meet its primary endpoint, patients showed improvements in multiple clinical manifestations and immunological measures of disease activity at week 24 [98].

#### 6.3.2. Anti-IFNα

Many SLE patients have a high expression of type 1 IFN-regulated genes in multiple cell types, also known as an IFN gene signature. Type 1 interferon signalling is mediated by the type 1 IFNα/β/ω receptor (IFNAR). In connection to SLE, TLR-7/9-mediated activation of pDCs increases the secretion of IFNα and leads to upregulation of BAFF/APRIL, which, in turn, activates B cells and autoreactive T cells. Also, type 1 IFN sensing by B cells decreases their threshold for BCR stimulation and also activates their antigen presentation capacity [104]. The initial attempt to inhibit IFNα using rontalizumab (binding human IFNα2) or sifalimumab failed [105,106]. These results shifted the focus to a blockage of the type 1 IFN receptor (IFNAR). Anifrolumab is a humanized monoclonal antibody against the type 1 interferon receptor subunit 1 (IFNAR-1). In a phase IIb study of anifrolumab, patients with active SLE who received the antibody intravenously during a 48-week period as an add-on to background standard SLE therapy showed sustained improvements in disease activity, assessed using the SLE Disease Activity Index 2000 (SLEDAI-2K) [107]. Even though the first phase III clinical trial (Treatment of Uncontrolled Lupus via the Interferon Pathway [TULIP]-1) showed efficacy across several components of the primary endpoint and key secondary endpoints, it failed to meet the composite primary endpoint of SRI-4 response at week 52 [108]. In a second phase III clinical trial (TULIP-2), 362 patients were enrolled and randomly assigned to the anifrolumab (300 mg) or placebo arm. In this trial, the primary endpoint was BICLA response at week 52, which is composed of a reduction in disease activity and no new worsening, based on the BILAG index, no worsening in the SLEDAI-2K, and no worsening greater than 10% in the Physician Global Assessment (PGA) (Table 2). The percentage of BICLA response in the anifrolumab and placebo groups was 47.8% and 31.5%, respectively. The treatment is now approved by the FDA in the US for the treatment of SLE, becoming the only novel biologic agent for lupus since belimumab came out [109].

#### 6.3.3. Anti-BTK

BTK is the key signalling molecule and kinase involved in the intracellular signalling of the B cell receptor. It is essential for B cell maturation, survival, and autoantibody production. Moreover, it is important for the activation of monocytes, macrophages, mast cells, and the release of IFNs. The selective inhibition of BTK in murine models of lupus has shown promise in treating skin lesions and lupus nephritis [101,110]. A recent multicentre phase II RCT of a selective BTK inhibitor, i.e., fenebrutinib (GDC-0853), showed that the overall safety profile of the drug was acceptable. However, patients who received fenebrutinib 150 mg once daily (*p* = 0.37) or 200 mg twice daily (*p* = 0.34) did not show a significant difference in SRI-4 response when compared with placebo at week 48 (Table 2) [99]. Due to the central role of BTK in B cell activation, BTK inhibitors provoked great interest in treating B cell-related malignancies and auto-immune diseases such as multiple sclerosis [111], immune thrombocytopenia [112], spontaneous urticaria [113], and chronic lymphocytic leukaemia [114]. Still, the success of BTK inhibitors for other diseases makes further studies on this type of treatment warranted.

#### 6.3.4. Anti-JAK

Both IFNα and IFNβ trigger a signalling cascade through the activation of the JAK/STAT pathway. In relation to B cell activation, it was found that JAK3 knockout mice have a deficit in B cell division, differentiation, and immunoglobulin production [115]. Tofacitinib is a JAK1/JAK3 inhibitor that targets this pathway by interfering with signalling from the common γ-chain used by several cytokine receptors. It was shown that tofacitinib ameliorated disease and reduced autoantibody titers in a murine model of lupus [116]. Also, tofacitinib has a direct effect on human B cells and blocks their differentiation to plasmablasts [117]. This treatment could also affect other pathways involved in B cell activation; to mention an example, Yan et al. found that IL-6 could downregulate TGFβR1 expression through the JAK/STAT3 pathway in T cells from SLE patients [118]. Thus, tofacitinib might affect disease activity by upregulating TGFβR1 expression and inhibiting T cell activation in SLE [118]. It has also shown an ability to decrease the expression of CD80/CD86 in LPS-stimulated DCs and decrease T cell co-stimulation in vitro, providing another mode of action for tofacitinib [119]. The phase I clinical trial of tofacitinib showed that a dose of tofacitinib (5 mg twice daily) is safe and well tolerated in SLE patients (Table 2) [100]. Tofacitinib improves atherosclerosis-related cardiometabolic parameters, as well as aberrant IFN-neutrophil interactions in SLE. Another JAK inhibitor that has been tested in SLE is baricitinib, an inhibitor of JAK1 and JAK2. The phase II trial of baricitinib (BRAVE I) assessed the effects of the 2 mg and 4 mg daily doses in active musculo-skeletal and cutaneous lupus; 68% of patients who received a dose of 4 mg baricitinib daily improved, based on the SLEDAI-2K (Table 2) [101]. Tyrosine kinase 2 (TYK2) is a newly discovered target in the JAK family for treating lupus. It is a non-receptor tyrosine kinase, which transduces IL-12, IL-23, and IFN signals. Since JAK family members are involved in many cytokine signalling pathways, multi-target JAK inhibitors usually are accompanied by dose-related safety concerns such as infections, lymphocytopenia, thromboembolism, and malignancies [120]. Deucravacitinib is a specific TYK2 inhibitor which inhibits TYK2 activation while not affecting the function of other JAK family targets. In a phase II, randomized, double-blind, placebo-controlled trial encompassing 363 patients with SLE, 58% vs. 34% of the patients who received deucravacitinib 3 mg twice daily vs. placebo, respectively, achieved an SRI-4 response (Table 2). Higher response frequencies were observed in the deucravacitinib 3 mg twice daily group at week 32 for BICLA response, skin-specific response based on the Cutaneous Lupus Erythematosus Disease Area and Severity Index 50 (CLASI-50), and attainment of Lupus Low Disease Activity State (LLDAS) [102]. In conclusion, JAK inhibitors, especially single-target JAK inhibitors, have yielded success in treating SLE and are currently being investigated further in multiple clinical trial phases.

#### 6.3.5. Anti-NIK

Since small molecule kinase inhibitors have the potential to broadly affect several signalling molecules, they have been developed in an effort to increase the specificity of their targeting [121]. One example is the NF-κB-inducing kinase (NIK), which is involved in the non-canonical NF-κB signalling from several TNF family receptors, including the BAFFR and TACI. Using mouse models of autoimmunity, Brightbill and colleagues treated the NZB/W F1 mice with an NIK inhibitor and showed that NIK inhibition could decrease OX40 and TWEAK signalling, improve survival, and reduce renal injury in the NZB/W F1 mice [122]. Interestingly, NIK inhibition also showed better responses and inhibition of autoantibody production compared with anti-BAFF treatment.

#### 6.3.6. Anti-Pin-1

Pin-1 is a prolyl-cis-trans-isomerase that is essential for TLR-7/TLR-9 signalling. After TLR-7/TLR-9 have been activated and bound to their ligands, Pin-1 mediates the nuclear translocation of IRF-7 and induction of IFNs. Pin-1 can also increase NF-κB activity by preventing NF-κB from binding to the endogenous inhibitory protein IκB. Genetic knockout of Pin-1 or use of a Pin-1 inhibitor attenuated the autoimmunity phenotype of lupus-prone mice [123]. Since the nucleic acid sensing TLR signalling has been shown to be important in disease development in all autoreactivities in murine lupus, it emerges as an attractive, more specific treatment. This approach could target the B cells that produce autoantibodies while permitting other B cells to respond normally [124].

### 6.4. Cell Therapy (CAR T Cell Therapy)

In recent years, anti-CD19 chimeric antigen receptor (CAR) T cell therapy gained some success in treating severe refractory SLE patients. The first case was a 20-year-old SLE patient with active disease whose SLEDAI score reached 16 before anti-CD19 CAR T cell therapy and was resistant to traditional treatment (hydroxychloroquine, systemic glucocorticoids, cyclophosphamide, MMF, tacrolimus, and belimumab) [125]. Autologous T cells transduced with a lentiviral anti-CD19 CAR vector (CD19 CAR T cells) were expanded in vitro and reinfused into the patient after lymphodepletion. The regimen of anti-CD19 CAR T therapy along with only low-dose steroids achieved full remission of disease at day 44 after treatment (SLEDAI score was 0). The anti-dsDNA antibodies titres and complement 3 and 4 levels also normalized within 5 weeks. The second attempt was in 2022 by Schett et al. [126]. They applied anti-CD19 CAR T cell therapy to 5 refractory SLE patients. Remission of SLE was achieved in all the patients and their SLEDAI score after 3 months was 0. The median drug-free remission time observed was 8 months after CAR T cell therapy. All of the patients showed normalized dsDNA antibodies and a dramatic decrease in other SLE-associated autoantibodies, meaning that the autoimmune processes were suppressed under CAR T cell therapy. The CAR T cell therapy was well-tolerated and only mild adverse events (cytokine-release syndrome and fever) appeared in those patients. In general, CAR T cell therapy showed better therapeutic efficiency over monoclonal antibodies that target B cells. This might be because CD19 CAR T cell therapy could deplete the autoreactive B cells within lymphatic organs and tissue where monoclonal antibody could not reach [127,128]. Another explanation is that CAR T cell therapy could target activated B cells, memory B cells, and plasma blasts, leading to a more complete depletion of B cells than with a CD20 monoclonal antibody or belimumab.

## 7. Future Perspectives

B cell-targeted therapy has made significant progress during the last few years and it is now possible to target B cells at different levels of their development and activation (Table 1 and Table 2). These therapies target either different B cells (including plasma cells, plasmablasts, mature B cells, and B cell precursors) or key signalling pathways for B cell maturation and activation. These enhanced treatment options may also lead to combinatory treatment regimens that can be tailored depending on the disease profile in the individual patient, and even on the phase of the disease. Thus, it is likely that it will be possible to apply more personalised medicine for SLE patients in the near future, utilising current and forthcoming drugs. As an increasing number of drugs become available, there will be a need to identify subgroups of patients that are expected to respond well to each one of these drugs or combinations thereof, by means of identification of those inflammatory pathways that dominate each phenotype, as well as the genetic make-up that is connected to the response to specific treatments or treatment combinations. It is also likely that to maximise efficacy, treatment regimens must be revisited to include the sequential use of drugs. Even though it might be too early to see how cell therapy can be applied in SLE, CD19-targeted CAR T cells recently showed merit for the treatment of refractory SLE, yielding remarkable immunological and clinical responses. It is imperative to gain better knowledge of how B cells function in connection with normal tolerance mechanisms, including their regulation and activation by the innate immune system.

## Figures and Tables

**Table 2:** A selection of clinical trials modifying cellular signalling and their endpoints of B cell-signalling targeted therapies for SLE.

Targets	Biologicals	Trial/Phase	Country	Number Enrolled	Primary Outcome	Result
CD40/CD40L	Dapirolizumab	Phase IIb (NCT02804763)	Europe, Latin America, and North America.	182	Dose–response based on BICLA responder rates at week 24	Did not meet the primary outcome but reduction in disease activity and severe flares was observed [98].
IFNAR	Anifrolumab	TULIP–2, Phase III (NCT02446899)	119 sites in 16 countries.	362	BICLA response at week 52	Met the primary outcome [99].
JAK1/3	Tofacitinib	Phase I (NCT02535689)	USA.	30	Safety of tofacitinib in SLE subjects (time frame: 5 years)	A dose of tofacitinib (5 mg twice daily) is safe and well tolerated in SLE patients [100].
JAK1/2	Baricitinib	BRAVE I, Phase II (NCT02708095)	78 centres in 11 countries in Asia, Europe, North America, and South America.	314	SLEDAI-2K arthritis or rash resolution at week 24	Met the primary outcome [101].
TYK2	Deucravacitinib	Phase II (NCT03252587)	162 sites in 17 countries in Asia, Europe, North America, and South America.	363	SRI-4 response at week 32	Met the primary outcome [102].
BTK	Fenebrutinib	Phase II (NCT02908100)	44 sites in 12 countries mainly in Latin America, the US, and Western Europe.	260	SRI-4 response at week 48	Did not meet the primary outcome, but significantly reduced levels of CD19-positive B cells, including plasmablasts [103].

BICLA: BILAG-based Composite Lupus Assessment; BTK: protein tyrosine kinase BTK; IFNAR: type I interferon receptor; SLEDAI-2K: Systemic Lupus Erythematosus Disease Activity Index 2000; SRI-4: SLE Responder Index 4; TYK2: Tyrosine Kinase 2.

## Data Availability

Not applicable.
